# Clinical Characteristics and Outcomes of Rome IV Diarrhea‐Predominant Irritable Bowel Syndrome and Functional Diarrhea Prescribed With Ondansetron: A Real‐World Study

**DOI:** 10.1111/nmo.70193

**Published:** 2025-11-02

**Authors:** Caterina Sbarigia, Christian Lambiase, Mohsin F. Butt, Abdulsalam I. Aliyu, Robin Spiller, Maura Corsetti

**Affiliations:** ^1^ NIHR Nottingham Digestive Diseases Centre, School of Medicine University of Nottingham Nottinghamshire UK; ^2^ Department of Medical‐Surgical Sciences and Translational Medicine Sant'Andrea Hospital, Sapienza University of Rome Rome Italy; ^3^ Gastrointestinal Unit, Department of Translational Research and New Technologies in Medicine and Surgery University of Pisa Pisa Italy; ^4^ Department of Molecular and Clinical Medicine Institute of Medicine, Sahlgrenska Academy, University of Gothenburg Gothenburg Sweden

**Keywords:** 5‐HT3 receptor antagonists, diarrhea‐predominant irritable bowel syndrome, disorders of gut‐brain interaction, functional diarrhea

## Abstract

**Background:**

5‐HT3 receptor antagonists, such as ondansetron, are effective in the management of irritable bowel syndrome‐diarrhea predominant (IBS‐D). However, real‐world data and evidence exploring the response to ondansetron in patients with functional diarrhea (FDr) are lacking. The aim of this study was to assess the clinical characteristics and response to ondansetron among patients with bowel disorders associated with chronic diarrhea in a real‐world setting.

**Methodology:**

We conducted a retrospective study of consecutive patients (October 2016–February 2024) diagnosed with IBS‐D or FDr in a tertiary care neurogastroenterology outpatient clinic. Demographic, clinical data, previous surgery, relevant investigations and current medications were collected. Clinical response to ondansetron was defined as (i) a reduction of at least one bowel movement/day versus baseline and (ii) firmer stool consistency (an improvement of ≥ 1 on the Bristol Stool Form Scale [BSFS] vs. baseline).

**Results:**

Ninety‐two patients were included, among whom 75 (81%) and 17 (18%) had IBS‐D and FDr, respectively. Urgency to defecate was significantly more frequent in patients with FDr versus IBS‐D (*p* = 0.003). Sixty‐eight of the 92 patients (73.9%) reported a clinical response to ondansetron, which was maintained at a median dosage of 8 mg/day for a median duration of 19 months with few minor side effects.

**Conclusion:**

When used in clinical practice, 8 mg/day of ondansetron is associated with a reduction in bowel frequency and an improvement in fecal consistency in approximately two‐thirds of patients with IBS‐D and FDr.

## Graphical abstract

In a cohort of 92 patients with chronic diarrhea (irritable bowel syndrome diarrhea predominant and functional diarrhea), nearly 74% of patients reported an improvement in their symptoms with ondansetron. Symptom relief was maintained long‐term with a low‐dose therapy.

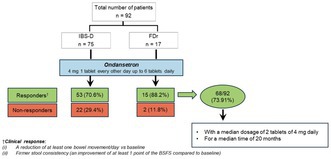

**Summary**

- In real‐world setting, 74% of patients with bowel disorders associated with chronic diarrhea (irritable bowel syndrome diarrhea predominant and functional diarrhea) reported a clinical improvement with ondansetron;
- A low dose ondansetron (median [IQR] 8 mg [8]) may be effective in patients with chronic diarrhea also in the long‐term;
- Treatment with ondansetron is associated with few minor side effects.

## 1 Introduction

Irritable bowel syndrome (IBS) and functional diarrhea (FDr) are the commonest disorders of gut‐brain interaction (DGBI) associated with chronic diarrhea [1, 2]. IBS diarrhea predominant (IBS‐D) is one of the four subtypes of IBS [3], each of which is classified according to the predominant stool form assessed by the Bristol Stool Form Scale (BSFS) [3, 4]. According to the Rome IV criteria, FDr is defined by the recurrent passage of loose or watery stools with symptoms that do not fulfill the criteria for IBS [3]. IBS‐D and FDr have a worldwide prevalence of 1.2% and 4.7%, respectively [1].

IBS‐D treatment includes antispasmodics and anti‐diarrheal medications as first‐line therapy, while second‐line therapies are represented by 5‐hydroxytryptamine receptor antagonists (5‐HT3‐RA), tricyclic antidepressants (TCA), eluxadoline, and occasionally bile acid sequestrants [2, 5, 6, 7]. More recently, other treatments such as medical devices have been demonstrated to be more effective than placebo in patients with IBS‐D [8].

The treatment of FDr is limited by the lack of evidence‐based studies on therapies specifically focused on this condition. Therefore, in clinical practice, therapies approved for IBS‐D are often used for treating FDr [2].

5‐HT3‐RA, such as ondansetron [9], alosetron [10] and ramosetron [11] have been demonstrated to be more effective than placebo agents in treating symptoms in IBS‐D. A recent network systematic review and meta‐analysis in patients with IBS‐D demonstrated that alosetron and ramosetron were highly effective in improving stool consistency and abdominal pain, respectively [12].

A recent phase III UK trial, that was supposed to confirm ondansetron efficacy and safety, did not meet its recruitment target because of the COVID‐19 pandemic, and failed to show a significant effect [9]. Therefore, ondansetron is not currently licensed as treatment for IBS‐D nor FDr in North America or Europe, despite the associated meta‐analysis showing overall effectiveness [9].

The aim of this real‐world study was to evaluate clinical characteristics and reported outcomes following treatment with ondansetron in patients with IBS‐D and FDr.

## 2 Materials and Methods

### 2.1 Design

In this retrospective study, data were collected from consecutive patients in a single tertiary care neurogastroenterology outpatient clinic (Queen's Medical Centre, Nottinghamshire, UK) between October 2016 and February 2024. The following inclusion criteria were applied: patients had to be aged ≥ 18 years, diagnosed with FDr or IBS‐D by the senior author (MC), prescribed ondansetron, and consulted at least twice during the timeframe. IBS‐D and FDr were diagnosed according to the clinical application of Rome IV criteria [13]. To make a positive diagnosis of IBS‐D and FDr, each patient was evaluated with limited investigations in line with national guidelines [7]. Briefly, coeliac screening and fecal calprotectin were performed in all the patients. A colonoscopy with biopsies was performed in patients with a fecal calprotectin level suggestive of inflammatory bowel disease as well as in those with suspected microscopic colitis. Bile acid malabsorption was considered in patients with risk factors, including but not limited to: those who had undergone a previous cholecystectomy, patients with a high body mass index, and/or severe diarrhea. When bile acid malabsorption was considered, a Selenium Homocholic Acid Taurine (SeHCAT) test was offered to patients. Patients diagnosed with Bile Acid Diarrhea (BAD) after performing a SeHCAT test were analyzed separately.

Data were systematically collected by the first author (CS) from clinic letters that were written using a standardized clinic template as previously reported [14, 15]. The latter was used to collect demographic and clinical data. Additional data related to physical and psychological co‐morbidities, previous abdominal or pelvic surgery, endoscopic procedures or other relevant investigations and current medications were collected. Each patient was started on ondansetron 4 mg every other day, with the possibility of titrating the dosage up to 24 mg per day according to clinical response. Clinical response was defined as (i) a reduction of at least one bowel movement/day versus baseline and (ii) firmer stool consistency (an improvement of at least 1 point on the BSFS compared to baseline). During each visit, patients were shown the BSFS chart, explained how it worked, and asked to indicate the most frequent type of stool consistency. This information was then recorded by the clinician in the patient's medical records.

In Nottinghamshire, an ondansetron prescription could not be re‐prescribed by general practitioners until the beginning of 2025. Therefore, repeated prescription was performed only by MC in those who had a positive response to treatment.

### 2.2 Statistical Analysis

The Shapiro–Wilk test was used to assess the normality of data. Continuous data are shown as median (interquartile range, IQR), whilst categorical variables are expressed as absolute and relative (%) frequency. Univariate analysis was performed using the Mann–Whitney *U* test for continuous variables and the Chi‐square test or Fisher's exact test for categorical variables, when appropriate. Pairwise comparisons were performed with Bonferroni correction for multiple testing. Reported *p*‐values are adjusted accordingly. For each test, a 2‐sided *p* value of ≤ 0.05 was considered significant. All statistical computations were performed using Jamovi software (Version 2.5.3.0).

## 3 Results

### 3.1 Patients

Ninety‐two patients were included: 81.5% (75/92) diagnosed with IBS‐D and 18.5% (17/92) with FDr. Clinical characteristics are shown in Table 1. The median age at the first consultation was 46.5 (25.3) years. Sixty‐seven percent (62/92) of patients were female. The median duration of symptoms before diagnosis was 4 (8) years. Patients were followed up for a median duration of 13.5 (20.5) months and for a median number of 2 (2) visits.

**TABLE 1:** Whole group characteristics at baseline.

**General characteristics**	
Total patients, *n*	92
IBS‐D, *n* (%)	69 (75)
Functional diarrhea, *n* (%)	16 (17.4)
Bile acid diarrhea, *n* (%)	7 (7.6)
Age at first consultation (years), median (IQR)	46.5 (25.3)
Female, *n* (%)	62 (67.4)
Duration of symptoms (years) before diagnosis, median (IQR)	4 (8)
Follow‐up time (months), median (IQR)	13.5 (20.5)
Follow‐up visits (number), median (IQR)	2 (2)
**Symptoms and comorbidities reported at first consultation**	
Bowel movements/daily^a^, median (IQR)	5 (6)
Stool consistency, *n* (%)	
*Bristol 5*	*31 (33.7)*
*Bristol 6*	*49 (53.3)*
*Bristol 7*	*12 (13)*
Fecal incontinence, *n* (%)	15 (16.3)
Bloating, *n* (%)	44 (47.8)
Urgency, *n* (%)	37 (40.2)
Nausea, *n* (%)	39 (42.4)
Dyspepsia, *n* (%)	21 (22.8)
Heartburn, *n* (%)	23 (25)
Dysphagia, *n* (%)	10 (10.9)
GI concomitant disorders, *n* (%)	19 (20.6)
Psychological disorders, *n* (%)	17 (18.5)
Previous abdominal surgery, *n* (%)	32 (34.8)
**Previous investigations**	
Gastroscopy, *n* (%)	36 (39.1)
Colonoscopy, *n* (%)	71 (77.2)
Colonoscopy with biopsies, *n* (%)	63 (68.5)
SeHCAT test, *n* (%)	31 (33.7)
Coeliac disease screening (TTG), *n* (%)	82 (89.1)
Fecal Calprotectin, *n* (%)	61 (65.6)
CRP, *n* (%)	68 (73.9)
**Medications**	
Loperamide, *n* (%)	34 (36.9)
Cholestyramine/colesevelam, *n* (%)	8 (8.7)
PPI, *n* (%)	24 (26.1)
Antispasmodics, *n* (%)	22 (23.9)
TCA/SSRI/SNRI/Tetracyclic, *n* (%)	35 (38)
Non‐opioid analgesics (paracetamol), *n* (%)	14 (15.2)
Opioids, *n* (%)	19 (20.6)
Metformin, *n* (%)	5 (5.4)

Abbreviations: CRP, C‐reactive protein; GI, gastro‐intestinal; IBS‐D, diarrhea‐predominant irritable bowel syndrome; PPI, proton pump inhibitors; SeHCAT, selenium homocholic acid taurine; SNRI, serotonin noradrenalyn receptors inhibitors; SSRI, serotonin selective receptors inhibitors; TCA, tricyclic antidepressants; TTG, tissue trans‐glutaminase.

^a^ On average.

### 3.2 Symptoms, Comorbidities and Investigations

At baseline, the median number of bowel movements (BMs) per day was five (six), and BSFS 5 [31 (33.7)] or 6 [49 (53.3)] were the most frequently reported stool form consistencies (Table 1).

Coeliac disease screening was performed with blood test in 89.1% of patients, while the remaining were screened by gastroscopy with duodenal biopsies. Seventy‐one patients (77.2%) had a colonoscopy and 63 (68.5%) of them underwent biopsies to exclude microscopic colitis. A positive fecal calprotectin value (above 50 μg/g) was found in 18 patients (29%) and only eight patients (44.4%) reported a value above 200 μg/g, but all of them had also a colonoscopy with biopsies that proved normal. A SeHCAT test was performed in 31 patients (33.7%). Seven (22.5%) of these (one with FDr and 6 with IBS‐D) were positive giving a diagnosis of BAD.

There were no differences between the three subgroups (IBS‐D, FDr and BAD) in terms of gender (*p* = 0.386) and number of BMs per day (*p* = 0.237) (Table 2).

**TABLE 2:** A comparison of baseline characteristics between patients with IBS‐D, FDr and BAD.

Baseline characteristics	IBS‐D (*n* = 69)	FDr (*n* = 16)	BAD (*n* = 7)	Group *p*	*p* IBS‐D versus FDr	*p* IBS‐D versus BAD	*p* FDr versus BAD
Gender, *n* (%)							
*Males*	25 (36.2)	4 (25)	1 (14.3)	0.386**	N/A	N/A	N/A
*Females*	44 (63.8)	12 (75)	6 (85.7)				
Bowel movements/daily, median (IQR)	5 (4)	5.5 (6.75)	10 (9.5)	0.237***	N/A	N/A	N/A
Stool consistency, *n* (%)							
*Bristol 5*	*24 (34.8)*	*5 (31.25)*	*2 (28.6)*	< 0.001**	0.003****	0.144****	1.00****
*Bristol 6*	*42 (60.9)*	*4 (25)*	*3 (42.8)*				
*Bristol 7*	*3 (4.3)*	*7 (43.75)*	*2 (28.6)*				
Symptoms, *n* (%)							
*Fecal incontinence*	*9 (13)*	*5 (31)*	*1 (14.3)*	*0.2* *	0.003****	0.6****	1.00****
*Bloating*	*35 (50.7)*	*6 (37.5)*	*3 (42.8)*	*0.535* *			
*Urgency*	*21 (30.4)*	*12 (75)*	*4 (57)*	*0.003* *			
*Nausea*	*32 (46.4)*	*5 (31.2)*	*2 (28.6)*	*0.4* *			
*Vomiting*	*10 (14.5)*	*2 (12.5)*	*1 (14.3)*	*0.979* *			
*Dyspepsia*	*16 (23.2)*	*3 (18.7)*	*2 (28.6)*	*0.866* *			
*Heartburn*	*18 (26)*	*2 (12.5)*	*3 (42.8)*	*0.685* *			
*Dysphagia*	*6 (8.7)*	*3 (18.7)*	*1 (14.3)*	*0.485* *			

Abbreviations: BAD, bile acid diarrhea; FDr, functional diarrhea; IBS‐D, diarrhea‐predominant irritable bowel syndrome.

* Fisher's exact test.

** Chi‐square test.

*** Kruskal‐Wallis test.

**** Bonferroni correction.

A lower proportion of patients with IBS‐D reported urgency versus patients with FDr (21 [30.4] vs. 12 [75], *p* = 0.001), while in terms of stool consistency, BSFS 6 was significantly more frequently reported in patients with IBS‐D compared to FDr (*p* = 0.003) (Table 2).

Notably, demographic and clinical characteristics among responders and non‐responders at baseline were overlapping and there were no significant differences in terms of stool frequency and consistency according to the BSFS (Table 3).

**TABLE 3:** Baseline characteristics in responders and non‐responders.

Baseline characteristics	Responders (*n* = 68)	Non‐responders (*n* = 24)	*p*
IBS‐D, *n* (%) FDr, *n* (%) BAD, *n* (%)	49 (71) 14 (87.5) 5 (71.4)	20 (29) 2 (12.5) 2 (28.6)	0.38*
Age, median (IQR)	46.5 (24.3)	43.5 (27.8)	0.5**
Female, *n* (%)	45 (66.2)	17 (70.8)	0.8*
Bowel movements/day, median (IQR)	5 (4)	6.5 (11.3)	0.46**
BSFS 6–7, *n* (%)	45 (66.2)	16 (66.7)	1*
Abdominal pain, *n* (%)	55 (80.9)	22 (91.7)	0.34*
Nausea, *n* (%)	28 (41.2)	11 (45.4)	0.81*
Vomiting, *n* (%)	12 (17.6)	1 (4.2)	0.17*
Urgency, *n* (%)	29 (42.5)	8 (33.3)	0.48*
Loperamide, *n* (%)	23 (67.6)	11 (32.4)	0.33*
Opioids, *n* (%)	12 (63)	7 (37)	0.25*
TCA, *n* (%)	11 (68.7)	5 (31.3)	0.75*

Abbreviations: BAD, bile acid diarrhea; BSFS, Bristol Stool Form Scale; FDr, functional diarrhea; IBS‐D, diarrhea‐predominant irritable bowel syndrome; TCA, tricyclic antidepressants.

* Fisher's exact test.

** Mann–Whitney's *U* Test.

### 3.3 Previous or Concomitant Medications

Regarding medications, 34 patients (36.9%) were previously on loperamide to treat diarrhea (Table 1). Of these, 15 patients (44.1%) stopped loperamide because of lack of efficacy, while four patients (11.7%) reported side effects (three patients reported constipation and one reported abdominal pain).

Bile acid binders (cholestyramine and colesevelam) were prescribed to eight patients (8.7%) with no benefits on diarrhea. Seven of these patients underwent a SeHCAT test and four individuals tested positive whilst three had normal results.

Thirty‐five patients (38%) were on concomitant neuromodulators. Nineteen patients (20.6%) were on concomitant opioids as long‐term treatment for non‐cancer pain, while non‐opioids analgesics (paracetamol) were consumed by 14 patients (15.2%) (Table 1).

### 3.4 Response to Ondansetron

Sixty‐eight patients (73.9%) responded to ondansetron. Among the responders, 49 (71%), 14 (87.5%), and five (71.4%) were respectively in the IBS‐D, FDr, and BAD subgroups. There was no difference in the proportion of patients who responded to ondansetron in the three groups (*p* = 0.38) (Table 3). Clinical response was maintained at a median dosage of eight (8) mg/daily, for a median follow‐up of 19 (20.3) months. Twenty‐four patients (26.1%) did not respond to treatment and terminated it.

**FIGURE 1:**
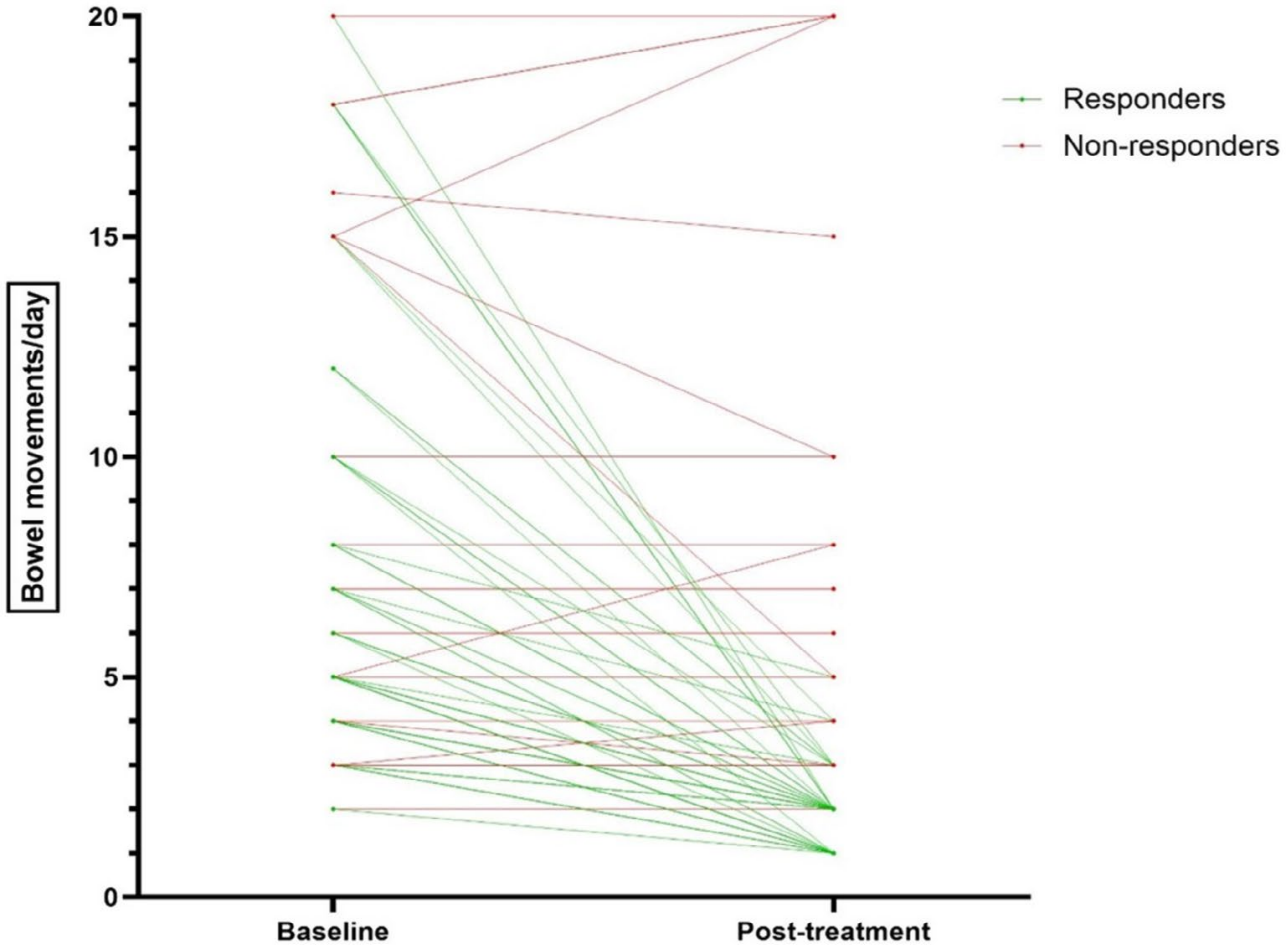
Changes in frequency of bowel movements at baseline and after treatment in responders and non‐responders.

Changes in the frequency of BMs are showed in Figure 1. Among responders (*n* = 68), the frequency of BMs dropped from a median baseline of five (4) to a median of two (1) per day after treatment.

**FIGURE 2:**
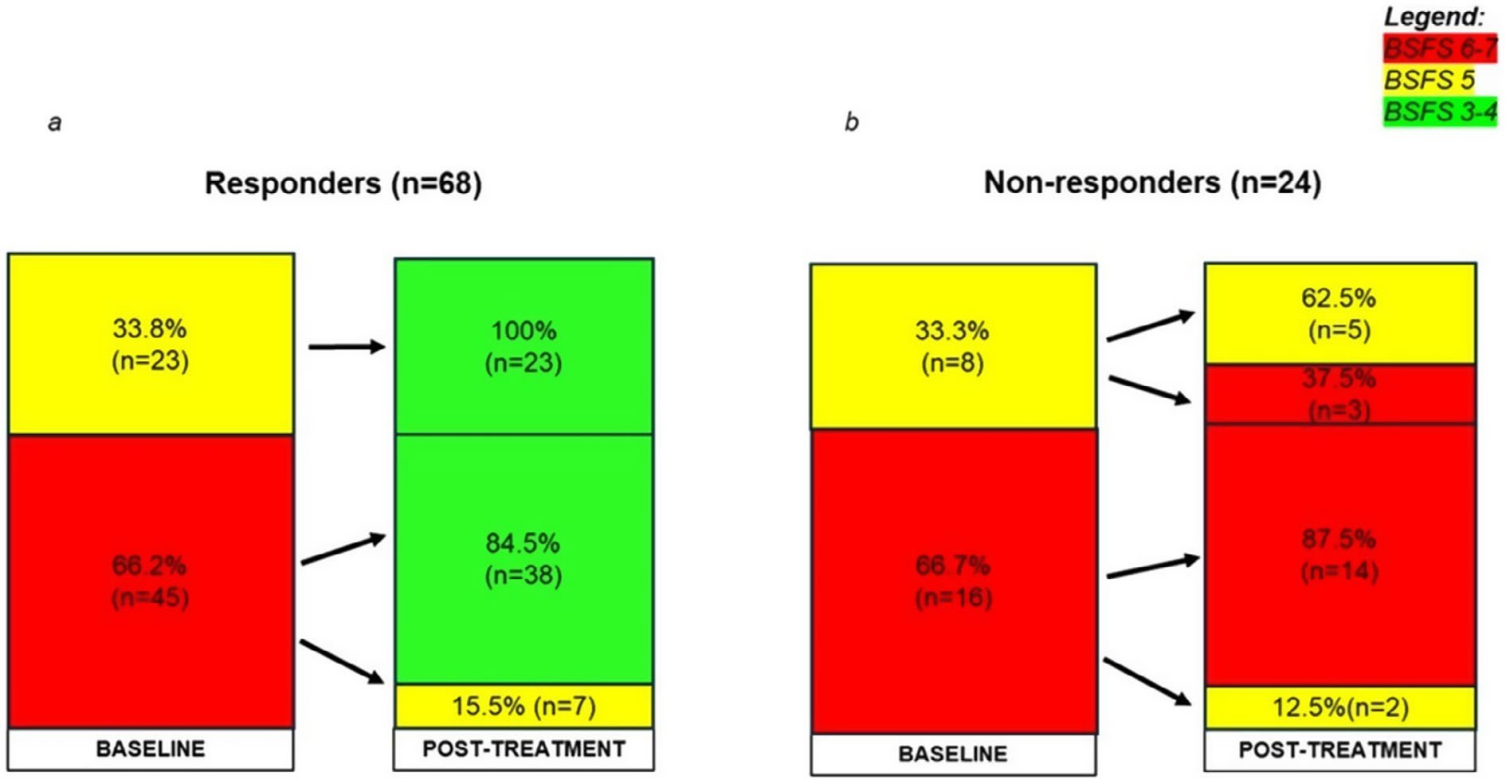
Stool consistency according to Bristol Stool Form Scale (BSFS) at baseline and post‐treatment in responders (a) and non‐responders (b).

Changes in stool consistency are showed in Figure 2a. Among responders (*n* = 68), forty‐five patients (66.2%) moved from a BSFS 6–7 to a BSFS 3–4 in 38 cases (84.5%) and to BSFS 5 in the remaining seven cases (15.5%), while the other 23 responders (33.8%) moved from BSFS 5 to a BSFS 3–4 after treatment (Figure 2a).

As shown in Figure 2b, among non‐responders (*n* = 24) sixteen patients (66.7%) had a BSFS 6–7 at baseline and only two of them (12.5%) moved to a BSFS 5 after treatment, but this was not associated with an improvement in the number of BMs per day, thus they were classified as non‐responders. Eight patients (33.3%) had a BSFS 5 at baseline and five of them showed no improvement on stool consistency after therapy, while three of them reported a worsening in stool consistency (BSFS 6) (Figure 2b).

Twelve patients out of the total (13%) had a BSFS 7 at baseline (3 IBS‐D, 7 FDr, and 2 BAD, respectively) and among them 11 patients (91.7%) responded to treatment.

In patients with IBS‐D, responders reported more frequently an improvement in abdominal pain compared to non‐responders [36 (73.5%) vs. 4 (18.2%), (*p* < 0.001)].

Side effects were reported in 22 patients (23.9%) (Table 4). The number of side effects was significantly higher in non‐responders compared to responders [10 vs. 12, (*p* = 0.02)], being constipation the most common one in both groups.

**TABLE 4:** Side effects in responders and non‐responders.

Side effects	Responders (*n* = 68)	Non‐responders (*n* = 24)	*p* *
Patients with side effects *n* = 22, *n* (%)	12 (17.6)	10 (41.7)	0.026
Constipation, *n* (%)	6 (8.8)	4 (16.7)	0.28
Abdominal pain, *n* (%)	2 (2.9)	3 (12.5)	0.10
Bloating, *n* (%)	1 (1.5)	0	1
Nausea, *n* (%)	2 (2.9)	3 (12.5)	0.1
Headache, *n* (%)	1 (1.5)	0	1
Not specified, *n* (%)	0	1 (4.2)	0.26

* Fisher's exact test.

## 4 Discussion

The present study evaluated the clinical characteristics and the response to ondansetron in patients diagnosed with chronic diarrhea in a clinical practice setting.

Approximately two‐thirds of patients responded to ondansetron in terms of both stool frequency and consistency and maintained the response at a median dosage of 8 mg/daily over a median follow‐up of 19 months. Ondansetron had a similar effect in IBS‐D and FDr patients. Notably, in patients with BSFS 7, response was achieved in more than 90% of patients.

These results suggest that patients with chronic diarrhea might benefit from a treatment with ondansetron, possibly achieving a clinical improvement with a low dosage of the medication, and maintaining the response in the long term with no major side effects. In this cohort, patients presented with a median of 5 BMs per day and more frequently with a BSFS of 5–6. Urgency and BSFS 7 were more frequently reported in the FDr group compared to IBS‐D. Overall, the most common symptoms associated with diarrhea were bloating, nausea and urgency, reported in 44 (47.8%), 39 (42.4%), and 37 (40.2%) patients, respectively. In addition, approximately one quarter of patients reported an overlap with heartburn or functional dyspepsia and about 20% of patients self‐reported psychological comorbidities. Overlapping comorbidities are frequent in patients with DGBIs [16] further contributing to lowering the quality of life of these patients [17].

As this study was uncontrolled, one cannot exclude the possibility that our results are due to a placebo effect. However, while a placebo response of around 34% can be expected for abdominal pain [18], a previous placebo‐controlled trial showed no placebo response for stool consistency [19], which seems a more objective measure. Thus, at least for stool consistency, the lack of a placebo in this report seems unlikely to distort the results.

As no placebo‐controlled RCT trials investigating the effects of ondansetron on FDr are currently available, our study represents the only indication that ondansetron might work also in this subgroup of patients with chronic diarrhea.

Interestingly, five out of the seven (71%) BAD patients achieved a clinical response to ondansetron, which was maintained for nearly 9 months. Three patients had a severe BAD (< 5% of retention) and achieved a response with an ondansetron dosage ranging from 12 mg to 24 mg daily, which is higher compared to the median of 8 mg effective in IBS‐D and FDr groups. We are aware that, although quite interesting, those data are not generalizable considering the low number of BAD cases, but our data suggest that this is worthy of future adequately powered, placebo‐controlled studies.

Constipation was the main side effect and it was reported by 10 patients. This is in line with previous studies [9, 19, 20, 21], and confirms the relatively favorable safety profile of the medication as compared to other drugs from the same class [22]. Our practice of titrating the dosage of ondansetron from 4 mg every other day to 8 mg three times/daily undoubtedly reduces the rate of severe constipation which was only 2% in our earlier RCT [19]. Therefore, in clinical practice, as in this study, patients are commonly suggested to adjust the dosage according to clinical response.

This study has several limitations, including its uncontrolled retrospective single‐centre design and the lack of standardized questionnaires for data collection. In addition, response to treatment was only assessed using patient‐reported outcomes. However, this is the first real‐world study with a similar sample size as previous randomized studies in IBS‐D [9, 19, 20, 21] but also including patients with FDr. Despite the absence of a placebo group, the present study supports the meta‐analysis which showed a stronger effect on stool consistency [Number Needed to Treat of 5] compared to the FDA composite endpoint (Number Needed to Treat = 9) [9]. While RCTs are necessary to confirm the efficacy and safety of medications before they could be approved by regulatory agencies, real‐world studies can be helpful to inform about the possible barriers in implementation in clinical practice. The present study suggests that ondansetron, prescribed as an off‐label medication, is likely to represent a helpful addition to the treatment of patients with chronic diarrhea.

In conclusion, ondansetron improved fecal consistency and reduced the frequency of bowel movements in patients with IBS‐D and FDr. At a median dosage of 8 mg, the effect of ondansetron appears to be durable in the long‐term with few side effects. Although promising, further data are needed to better assess its efficacy and to regulate its use in chronic diarrhea‐related disorders.

## Author Contributions

C.S. collected data, took the lead in writing the manuscript and performed data analysis. C.L. and M.F.B. assisted in data analysis and reviewed the manuscript. A.I.A. assisted in data collection and analysis. M.C. and R.S. reviewed the manuscript and supervised the project. All authors read and approved the final manuscript.

## Ethics Statement

This study was performed in line with the principles of the Declaration of Helsinki.

## Conflicts of Interest

R.S. has received research grants from Nestle and Sanofi and is a consultant for EnteroBiotix. M.C. is a consultant for Mayoly, PROMEDCS, Biocodex, and is a co‐investigator in a research study funded by Sanofi and another funded by Nestle. All other authors declare no conflicts of interest.

## Data Availability

The data that support the findings of this study are available from the corresponding author upon reasonable request.

## References

[1] A. D. Sperber , S. I. Bangdiwala , D. A. Drossman , et al., “Worldwide Prevalence and Burden of Functional Gastrointestinal Disorders, Results of Rome Foundation Global Study,” Gastroenterology 160, no. 1 (2021): 99–114.e3, 10.1053/j.gastro.2020.04.014. 32294476

[2] E. Savarino , F. Zingone , B. Barberio , et al., “Functional Bowel Disorders With Diarrhoea: Clinical Guidelines of the United European Gastroenterology and European Society for Neurogastroenterology and Motility,” United European Gastroenterology Journal 10, no. 6 (2022): 556–584, 10.1002/ueg2.12259. 35695704 PMC9278595

[3] B. E. Lacy , F. Mearin , L. Chang , et al., “Bowel Disorders,” Gastroenterology 150, no. 6 (2016): 1393–1407.e5, 10.1053/j.gastro.2016.02.031. 27144627

[4] S. J. Lewis and K. W. Heaton , “Stool Form Scale as a Useful Guide to Intestinal Transit Time,” Scandinavian Journal of Gastroenterology 32, no. 9 (1997): 920–924, 10.3109/00365529709011203. 9299672

[5] A. J. Lembo , B. E. Lacy , M. J. Zuckerman , et al., “Eluxadoline for Irritable Bowel Syndrome With Diarrhea,” New England Journal of Medicine 374, no. 3 (2016): 242–253, 10.1056/NEJMoa1505180. 26789872

[6] G. Barbara , C. Cremon , M. Bellini , et al., “Italian Guidelines for the Management of Irritable Bowel Syndrome: Joint Consensus From the Italian Societies of: Gastroenterology and Endoscopy (SIGE), Neurogastroenterology and Motility (SINGEM), Hospital Gastroenterologists and Endoscopists (AIGO), Digestive Endoscopy (SIED), General Medicine (SIMG), Gastroenterology, Hepatology and Pediatric Nutrition (SIGENP) and Pediatrics (SIP),” Digestive and Liver Disease 55, no. 2 (2023): 187–207, 10.1016/j.dld.2022.11.015. 36517261

[7] D. H. Vasant , P. A. Paine , C. J. Black , et al., “British Society of Gastroenterology Guidelines on the Management of Irritable Bowel Syndrome,” Gut 70, no. 7 (2021): 1214–1240, 10.1136/gutjnl-2021-324598. 33903147

[8] C. A. Howell , A. Kemppinen , V. Allgar , et al., “Double‐Blinded Randomised Placebo Controlled Trial of Enterosgel (Polymethylsiloxane Polyhydrate) for the Treatment of IBS With Diarrhoea (IBS‐D),” Gut 71, no. 12 (2022): 2430–2438, 10.1136/gutjnl-2022-327293. 35760493 PMC9664110

[9] D. Gunn , R. Topan , L. Barnard , et al., “Randomised, Placebo‐Controlled Trial and Meta‐Analysis Show Benefit of Ondansetron for Irritable Bowel Syndrome With Diarrhoea: The TRITON Trial,” Alimentary Pharmacology & Therapeutics 57, no. 11 (2023): 1258–1271, 10.1111/apt.17426. 36866724

[10] M. Camilleri , W. Y. Chey , E. A. Mayer , et al., “A Randomized Controlled Clinical Trial of the Serotonin Type 3 Receptor Antagonist Alosetron in Women With Diarrhea‐Predominant Irritable Bowel Syndrome,” Archives of Internal Medicine 161, no. 14 (2001): 1733–1740. 11485506 10.1001/archinte.161.14.1733

[11] K. Matsueda , S. Harasawa , M. Hongo , N. Hiwatashi , and D. Sasaki , “A Randomized, Double‐Blind, Placebo‐Controlled Clinical Trial of the Effectiveness of the Novel Serotonin Type 3 Receptor Antagonist Ramosetron in Both Male and Female Japanese Patients With Diarrhea‐Predominant Irritable Bowel Syndrome,” Scandinavian Journal of Gastroenterology 43, no. 10 (2008): 1202–1211, 10.1080/00365520802240255. 18618371

[12] C. J. Black , N. E. Burr , M. Camilleri , et al., “Efficacy of Pharmacological Therapies in Patients With IBS With Diarrhoea or Mixed Stool Pattern: Systematic Review and Network Meta‐Analysis,” Gut 69, no. 1 (2020): 74–82, 10.1136/gutjnl-2018-318160. 30996042

[13] D. A. Drossman and J. Tack , “Rome Foundation Clinical Diagnostic Criteria for Disorders of Gut‐Brain Interaction,” Gastroenterology 162, no. 3 (2022): 675–679, 10.1053/j.gastro. 34808139

[14] M. F. Butt , F. Cefalo , C. Sbarigia , A. Dhali , and M. Corsetti , “Impact of Opioid and Cannabis Use on Low‐Dose Amitriptyline Efficacy in Cyclical Vomiting Syndrome: A Real‐World Study in the United Kingdom,” Neurogastroenterology and Motility 37, no. 6 (2025): e70007, 10.1111/nmo.70007. 40017095 PMC12075911

[15] M. F. Butt , G. Isherwood , T. Lewis‐Lawson , et al., “Clinical Characteristics and Outcomes of Patients With Rome IV Functional Dyspepsia Who Consume Opioids: A Real‐World Study,” Neurogastroenterology and Motility 37, no. 7 (2025): e15019, 10.1111/nmo.15019. 40017096 PMC12163204

[16] I. Aziz , O. S. Palsson , H. Törnblom , A. D. Sperber , W. E. Whitehead , and M. Simrén , “The Prevalence and Impact of Overlapping Rome IV‐Diagnosed Functional Gastrointestinal Disorders on Somatization, Quality of Life, and Healthcare Utilization: A Cross‐Sectional General Population Study in Three Countries,” American Journal of Gastroenterology 113, no. 1 (2018): 86–96, 10.1038/ajg.2017.421. 29134969

[17] N. A. Koloski , M. Jones , and N. J. Talley , “Evidence That Independent Gut‐To‐Brain and Brain‐To‐Gut Pathways Operate in the Irritable Bowel Syndrome and Functional Dyspepsia: A 1‐Year Population‐Based Prospective Study,” Alimentary Pharmacology & Therapeutics 44, no. 6 (2016): 592–600, 10.1111/apt.13738. 27444264

[18] M. Bosman , S. Elsenbruch , M. Corsetti , et al., “The Placebo Response Rate in Pharmacological Trials in Patients With Irritable Bowel Syndrome: A Systematic Review and Meta‐Analysis,” Lancet Gastroenterology & Hepatology 6, no. 6 (2021): 459–473, 10.1016/S2468-1253(21)00023-6. 33765447

[19] K. Garsed , J. Chernova , M. Hastings , et al., “A Randomised Trial of Ondansetron for the Treatment of Irritable Bowel Syndrome With Diarrhoea,” Gut 63, no. 10 (2014): 1617–1625, 10.1136/gutjnl-2013-305989. 24334242 PMC4173656

[20] T. F. Plasse , G. Barton , E. Davidson , et al., “Bimodal Release Ondansetron Improves Stool Consistency and Symptomatology in Diarrhea‐Predominant Irritable Bowel Syndrome: A Randomized, Double‐Blind, Trial,” American Journal of Gastroenterology 115, no. 9 (2020): 1466–1473, 10.14309/ajg.0000000000000727. 32639235

[21] S. Jafari , A. Atmani , S. Gohari , and E. Seifi , “The Effect of Ondansetron on Improvement of Symptoms in Patients With Irritable Bowel Syndrome With Diarrhea Domination: A Randomized Controlled Trial,” Middle East Journal of Digestive Diseases 16, no. 3 (2024): 178–184, 10.34172/mejdd.2024.386. 39386337 PMC11459287

[22] L. Chang , W. D. Chey , L. Harris , K. Olden , C. Surawicz , and P. Schoenfeld , “Incidence of Ischemic Colitis and Serious Complications of Constipation Among Patients Using Alosetron: Systematic Review of Clinical Trials and Post‐Marketing Surveillance Data,” American Journal of Gastroenterology 101, no. 5 (2006): 1069–1079, 10.1111/j.1572-0241.2006.00459.x. 16606352

